# Bayesian Inference of Sampled Ancestor Trees for Epidemiology and Fossil Calibration

**DOI:** 10.1371/journal.pcbi.1003919

**Published:** 2014-12-04

**Authors:** Alexandra Gavryushkina, David Welch, Tanja Stadler, Alexei J. Drummond

**Affiliations:** 1 Department of Computer Science, University of Auckland, Auckland, New Zealand; 2 Allan Wilson Centre for Molecular Ecology and Evolution, Massey University, Palmerston North, New Zealand; 3 Department of Biosystems Science and Engineering, ETH Zürich, Switzerland; Duke University, United States of America

## Abstract

Phylogenetic analyses which include fossils or molecular sequences that are sampled through time require models that allow one sample to be a direct ancestor of another sample. As previously available phylogenetic inference tools assume that all samples are tips, they do not allow for this possibility. We have developed and implemented a Bayesian Markov Chain Monte Carlo (MCMC) algorithm to infer what we call sampled ancestor trees, that is, trees in which sampled individuals can be direct ancestors of other sampled individuals. We use a family of birth-death models where individuals may remain in the tree process after sampling, in particular we extend the birth-death skyline model [Stadler *et al.*, 2013] to sampled ancestor trees. This method allows the detection of sampled ancestors as well as estimation of the probability that an individual will be removed from the process when it is sampled. We show that even if sampled ancestors are not of specific interest in an analysis, failing to account for them leads to significant bias in parameter estimates. We also show that sampled ancestor birth-death models where every sample comes from a different time point are non-identifiable and thus require one parameter to be known in order to infer other parameters. We apply our phylogenetic inference accounting for sampled ancestors to epidemiological data, where the possibility of sampled ancestors enables us to identify individuals that infected other individuals after being sampled and to infer fundamental epidemiological parameters. We also apply the method to infer divergence times and diversification rates when fossils are included along with extant species samples, so that fossilisation events are modelled as a part of the tree branching process. Such modelling has many advantages as argued in the literature. The sampler is available as an open-source BEAST2 package (https://github.com/CompEvol/sampled-ancestors).

## Author Summary

A central goal of phylogenetic analysis is to estimate evolutionary relationships and the dynamical parameters underlying the evolutionary branching process (e.g. macroevolutionary or epidemiological parameters) from molecular data. The statistical methods used in these analyses require that the underlying tree branching process is specified. Standard models for the branching process which were originally designed to describe the evolutionary past of present day species do not allow one sampled taxon to be the ancestor of another. However the probability of sampling a direct ancestor is not negligible for many types of data. For example, when fossil and living species are analysed together to infer species divergence times, fossil species may or may not be direct ancestors of living species. In epidemiology, a sampled individual (a host from which a pathogen sequence was obtained) can infect other individuals after sampling, which then go on to be sampled themselves. The models that account for direct ancestors produce phylogenetic trees with a different structure from classic phylogenetic trees and so using these models in inference requires new computational methods. Here we developed a method for phylogenetic analysis that accounts for the possibility of direct ancestors.

## Introduction

Phylogenetic analysis uses molecular sequence data to infer evolutionary relationships between organisms and to infer evolutionary parameters. Since the introduction of Bayesian inference in phylogenetics [1]–[3], it has become the standard approach for fully probabilistic inference of evolutionary history with many popular implementations [4]–[7] of Markov chain Monte Carlo (MCMC) [8], [9] sampling over the space of phylogenetic trees. Initial descriptions of Bayesian phylogenetic analysis were restricted to considering bifurcating trees [1], [2], but have been extended to include explicit polytomies [10]. Here we tackle phylogenetic inference with trees that may contain sampled ancestors [11].

Standard phylogenetic models developed for inferring the evolutionary past of present day species assume that all samples are terminal (leaf) nodes in the estimated phylogenetic tree. However, serially sampled data generated by different evolutionary processes can be analysed using phylogenetic methods [12] and, in some cases, the assumption that all sampled taxa are leaf nodes is not appropriate.

One case in point is when inferring epidemiological parameters from viral sequence data obtained from infected hosts [13]–[17]. Viral sequences are obtained from distinct hosts and treated as samples from the transmission process. Using standard phylogenetic models (such as coalescent or birth-death models) to describe the infectious disease transmission process entails the assumption that a host becomes uninfectious at sampling (where sampling is obtaining a sequence or sequences from the pathogen population residing in a single infected host). However in many cases, hosts remain infectious after sampling and, when sampling is sufficiently dense, the probability of sampling an individual that later infects another individual which is also sampled is not negligible [18]–[20].

A recent analysis of a well-characterised HIV transmission chain [20] employed a hierarchical model of a gene tree inside a transmission tree to infer the differences in evolutionary rates (substitution rates) within and among hosts. Hierarchical modelling of gene trees inside transmission trees has also been used to infer transmission events for small epidemic outbreaks where epidemiological data is available in the form of known infection and recovery times for each host [16]. In both cases the inference of transmission trees assumes complete sampling of the hosts involved, and the host sampling process is not explicitly modelled.

Incomplete sampling is explicitly accounted for by birth-death-sampling models [15], [21]–[23], and the probability density functions of the trees are available in closed form, thus making these models tractable for use in Bayesian inference. The birth-death-sampling models do not assume that individuals are removed from the tree process after the sampling. However, using models that allow for infection after sampling has not been possible due to a lack of software, meaning that many analyses simply ignore the possibility of sampled ancestors [15], [23].

Another problem that may require sampled ancestor models is inferring species divergence times using fossil data. Without the means to calibrate the times of divergences, the length of branches in the estimated molecular phylogeny of contemporaneous sequences are typically described in units of expected substitutions per site. Geologically dated fossil data can be employed to calibrate a phylogenetic tree, thus providing absolute branch lengths in calendar units. The most common approach here is to specify age limits or a probability density function on specific divergence times in the phylogeny, where the constraints are defined using the fossil data [24]–[28]. There are several drawbacks connected to this approach [29], [30]. First, there is potential for inconsistency when applying two priors on the phylogeny [31]: a calibration prior on one or more divergence times and a tree process prior on the entire tree. Second, it is not obvious how to specify a calibration density so that it accurately reflects prior knowledge about divergence times [29], [30]. Finally, such densities usually only use the oldest fossil within a particular clade, thus discarding much of the information available in the fossil record [30].

Other methods for dating with fossils have been developed recently [32]. One approach that addresses the problems of the node calibration method requires modelling fossilisation events as a part of the tree process prior. This allows for the joint analysis of fossil and recent taxa together in a unified framework [29], [30], [33]–[36]. Models that jointly describe the processes of macroevolution and fossilisation should account for possible ancestor-descendant relationships between fossil and living species [37], and thus include sampled ancestors.

Wilkinson and Tavaré [38] used the inhomogeneous birth-death process with sampled ancestors and approximate Bayesian computation methods to estimate divergence times from fossil records and known features of the extant phylogeny. A birth-death model with sampled ancestors has been used to estimate speciation and extinction rates from phylogenies in [22]. Heath *et al.* [30] have used this model (they call it the *fossilized birth-death process*) to explicitly model fossilisation events and estimate divergence times from molecular data and fossil records in a Bayesian framework. In their approach, the tree topology relating the extant species has to be known for the inference [30]. So a method that simultaneously estimates the divergence times and tree topology while modelling incorporation of sampled fossil taxa is an obvious next step.

Full Bayesian MCMC inference using models with sampled ancestors is complicated by the fact that such models produce trees, which we call *sampled ancestor trees* [11], that are not strictly binary. They may have sampled nodes that lie on branches, forming an internal node with one direct ancestor and one direct descendent. Thus, modelling sampled ancestors induces a tree space where the tree has a variable number of dimensions (a function of the number of sampled ancestors), which necessitates extensions to the standard MCMC tree sampling algorithms.

Here we describe a reversible-jump MCMC proposal kernel [39] to effectively traverse the space of sampled ancestor trees and implement it within the BEAST2 software platform [6]. We study the limitations of birth-death models with sampled ancestors and extend the birth-death skyline model [23] to sampled ancestor trees. We apply the new posterior sampler to two types of data: a serially sampled viral data set (from HIV), and molecular phylogeny of bear sequences with fossil samples.

## Methods

### Tree models with sampled ancestors

In this section, we consider birth-death sampling models [15], [21]–[23] under the assumption that sampled individuals are not necessarily removed from the process at sampling. This results in a type of phylogenetic tree that may contain degree two nodes called *sampled ancestors*.

**Figure 1:**
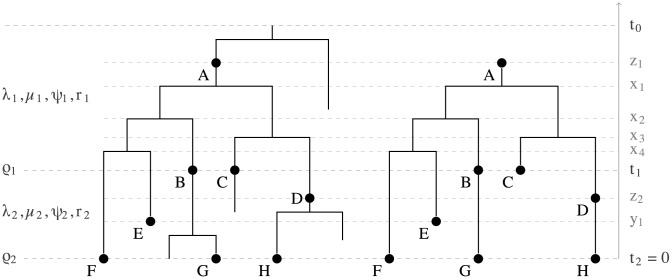
Full tree versus reconstructed tree. A full tree produced by the sampled ancestor birth-death process on the left and a reconstructed tree on the right. The sampled nodes are indicated by dots labeled by letters A through H. Nodes A, B and D are sampled ancestors. The reconstructed tree is represented by a sampled ancestor tree 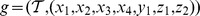, where 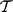 denotes the ranked tree topology and 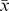, 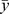, and 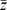 denote the node ages. In the reconstructed tree the root is a sampled node. In the skyline model, birth-death parameters vary from interval to interval. There are two intervals in this figure bounded by the time of origin 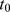, parameter shift time 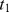, and present time 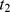. Between 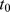 and 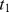 parameters 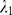, 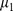, 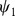 and 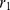 apply and between 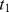 and 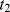 parameters 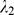, 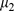, 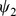, and 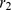. There are additional sampling attempts at times 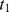 and 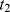 with sampling probabilities 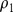 and 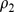.

An important characteristic of the models we consider here is incomplete sampling, i.e., we only observe a part of the tree produced by the process. Consider a birth-death process that starts at some point in time (the time of origin) with one lineage and then each existing lineage may bifurcate or go extinct. Further, the lineages are randomly sampled through time. An example of *a full tree* produced by such process is shown in Figure 1 on the left. We have information only about the portion of the process that produces the samples, shown as labeled nodes, and do not observe the full tree. Thus we only consider this subtree relating to the sample, which is called the *reconstructed tree* (or the *sampled tree*) and is shown on the right of Figure 1.

#### The sampled ancestor birth-death model

Here we describe a serially-sampled birth-death model with sampled ancestors [15], [21]. First we describe a variant of the model suited to modelling transmission processes and then we extend the model to describe speciation and fossilization processes.

The process begins at the time of origin 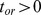 measured in time units before the present. Moving towards the present, each existing lineage bifurcates or goes extinct according to two independent Poisson processes with constant rates 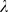 and 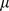, respectively. Concurrently, each lineage is sampled with Poisson rate 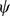 and is removed from the process at sampling with probability 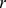. The process is stopped at time 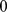. This process can be used to model the transmission of infectious disease and we call it *the transmission birth-death process*.

The transmission process involves sampling individuals and produces trees that have degree two nodes corresponding to sampling events when a lineage was sampled but was not removed. We call these trees *sampled ancestor trees* (whether or not any sampled ancestors are present). The reconstructed tree has degree-two nodes when a lineage is sampled but not removed and then it, or a descendent lineage, is sampled again. The reconstructed tree in Figure 1 (on the right) is an example of a sampled ancestor tree. Note that the root of a sampled ancestor tree is the most recent common ancestor of the sampled nodes and therefore it may be a sampled node. There is no origin node in the tree because the time of origin is a model parameter and not an outcome of the process.

A tree (or genealogy) 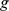 consists of the discrete component 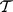, which is called *a tree topology*, and the continuous component 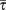, which is called *a time vector*. The tree topology of a sampled ancestor tree is *a sampled ancestor phylogenetic tree*, which is a ranked labeled phylogenetic tree with labeled degree-two vertices (a rigorous definition of a sampled ancestor phylogenetic tree can be found in [11], where it is called an FRS tree). The time vector is a real-valued vector of the same dimension as the number of ranks (nodes) in the tree topology and with coordinates going in the descending order so that each node in the tree topology can be unambiguously assigned a time from the time vector.

Further, we have three types of nodes: bifurcation nodes, sampled tip nodes, sampled internal nodes. Let 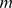 be the number of leaves, then 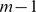 is the number of bifurcation events. Let 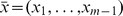 be a vector of bifurcation times, where 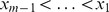. Let 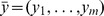 be a vector of tip times, where 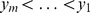. Further let 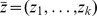 be a vector of times of sampled two degree nodes, where 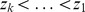 and 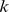 is the number of such nodes. Then 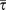 can be obtained by combining elements of 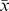, 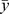, and 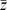 and ordering them in the descending order (see also Figure 1). A genealogy may be written as 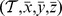.

Stadler *et al.* [15] derived the density of a genealogy 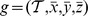 given the transmission birth-death process parameters 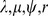 and time of origin 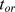. In [21], it was indicated that we should also condition on the event, 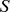, of sampling at least one individual because only non-empty samples are observed. The density is
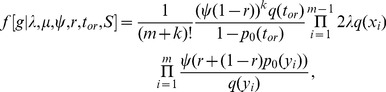
(1)where the function 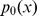 is the probability that an individual has no sampled descendants for a time span of length 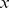 so that 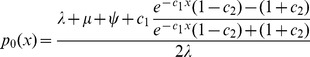
where 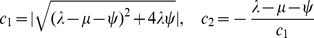
and 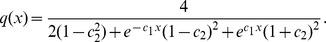
Throughout this paper, we consider non-oriented labeled trees (in oriented trees, each non-root node is labeled as the left or right child of its parent). So equation (1) differs from the equation on page 350 in [15], written for oriented trees, by a factor accounting for the switch from oriented to labeled trees and also by the term for conditioning on 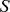. Note also that the definition of the function 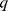 here is different from the definition in [15].

We show in the Supporting Information (Theorem 2 in Text S1) that function (1) depends only on three parameters: 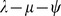, 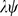, and 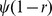, and does not depend on parameters 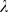, 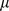, 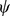 and 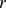 independently. This means that the tree model is unidentifiable but, as we show in simulation studies, if we specify one of the parameters we can estimate the others.

When applying this model to data, we typically shift time such that the most recent tip occurs at present, 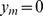, as we often do not have information about the length of time between the last sample and the end of the sampling effort. This is done to reduce our set of unknown quantities by one (namely setting 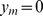).

We extend the model to allow the possibility of sampling individuals at present, where each lineage at time 0 is sampled with probability 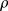. This process, with 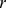 set to zero (which implies that an individual is not removed from the process after sampling) can be used to model speciation processes with fossilisation events, hence it is called *the fossilized birth-death process* [30]. Let 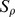 denote the event of sampling at least one individual at present then according to [21] and accounting for labeled trees: 
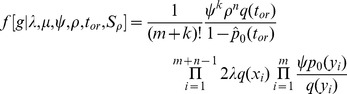
(2)where 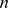 is the number of 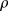-sampled tips, 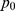, 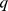 and 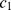 defined as above with 
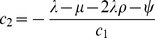
and 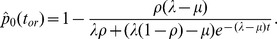
In contrast to the transmission birth-death process, where only three out of the four parameters 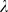, 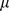, 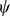, and 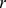 can be inferred, under the fossilized birth-death process, all four parameters 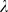, 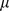, 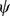, and 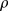 can be identified from the phylogeny as we show in simulation studies.

It is possible to re-write density (2) conditioning on the time of the most recent common ancestor of sampled individuals rather than conditioning on the time of origin. In this case, we discard trees in which the root is a sampled node. In other words, we assume that the process starts with a bifurcation event and we only consider trees with sampled nodes on both sides of the initial bifurcation event. Then the time of the most recent common ancestor of the sample is the time of the root, 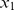. Accounting for labeled trees, the probability density function can thus be written [21] as:
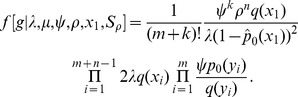
(3)where 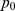, 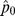, and 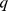 are defined as in equation (2).

The probability of an individual sampled at time 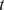 before present to be a sampled ancestor is 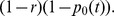
Thus, the fact that an individual is a sampled ancestor depends on whether the individual stays in the process after it is sampled or not (determined by 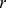), the rate of population growth (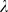 and 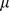), sampling rates (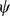 and 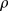) and the amount of time (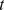) elapsed until present. If the population grows fast and/or the sampling rate is high and/or the amount of time elapsed is large then the probability of an individual sampled at time 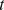 (before present) leaving sampled descendants is high.

#### The sampled ancestor skyline model

Here we extend the sampled ancestor birth-death model so that parameters may change through time in a piecewise manner. This model combines two models from [15] and [23].

Let there be 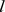 time intervals 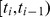 for 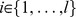 defined by vector 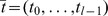 and 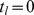 with 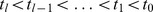 (where 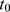 plays the role of the origin time, i.e., 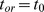). We use notation 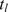 for time zero only for convenience and do not include it as a model parameter. Within each interval 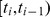, 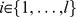 the constant birth-death parameters 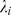, 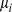, 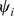, and 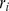 apply. At the end of each interval at times 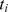, 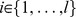, each individual may be sampled with probability 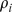 (see also Figure 1). Thus, the model has 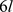 parameters: 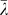, 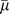, 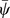, 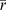, 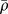, and 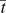. We prove in the Supporting Information (Theorem 1 in Text S1) that the probability density of a reconstructed sampled ancestor tree 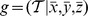 produced by this process is (not conditioned on survival), 
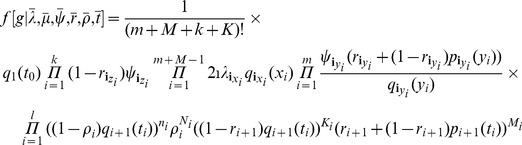
(4)where 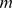 is the number of 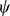-sampled tips; 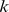 is the number of 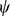-sampled nodes that have sampled descendants; 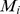 is the number of tips sampled at time 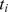; 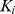 is the number of nodes sampled at time 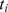 and having sampled descendants; 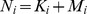 is the total number of nodes sampled at time 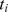; 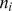 is the number of lineages present in the tree at time 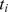 but not sampled at this time for 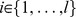; 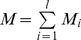; 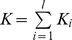; 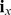 is an index such that 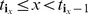; and functions 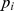 and 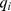 are defined presently.

The probability 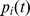 that an individual alive at time 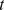 has no sampled descendants when the process is stopped (i.e., in the time interval 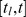), with 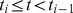 (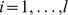) is 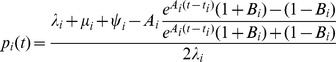
where 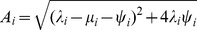
and 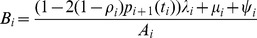
for 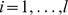 and 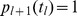. Further, 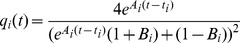
for 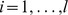. Note that 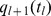 does not appear in the equation because 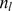 (which is the number of lineages present in the tree at time 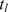 but not sampled at that time) and 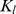 (which is the number of two degree nodes at time 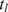) are always zero. Also, 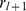 cancels out because 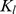 is always zero and 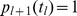.

We obtain two special cases of this general model that correspond to the skyline variants of the transmission and fossilized birth-death processes by setting some of the parameters to zero.

To obtain the skyline transmission process, we set 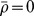. This implies 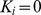, 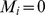, and 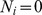 for all 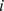. As before, we condition on the event, 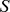, of sampling at least one individual, where 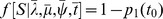. The tree density is 
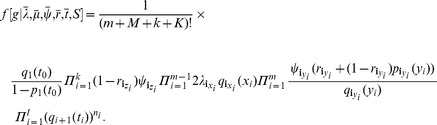
(5)We show in the Supporting Information (Theorem 2 in Text S1) that (5) can be re-parameterised with 
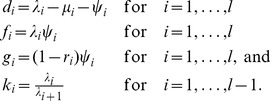
(6)Thus, of the original 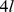 parameters, only 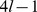 may be estimated.

For the skyline fossilized birth-death model, we set 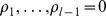 and 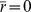 and condition on 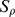, the event of sampling at least one extant individual (i.e., at time 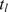). The tree density becomes
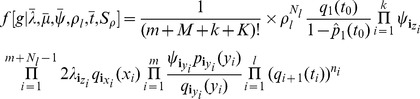
(7)where 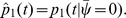
This probability density can be re-parameterised as in (6) with one additional equation 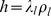 (see Text S1). Now there are 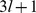 initial parameters: 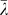, 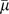, 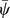, and 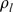 and 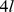 equations defining the re-parameterisation. Since 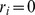, 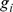 defines 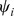, then 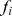 yields 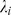, then 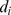 yields 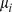, 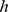 yields 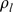 and the 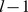 equations for 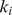 are not needed at all, thus 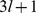 equations define the re-parameterisation of the 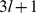 parameters and this re-parameterisation does not reduce the number of parameters.

### Markov chain Monte Carlo operators

We introduce a number of operators to explore the space of sampled ancestor trees with a fixed number of sampled nodes. Throughout this section, we denote the height (or the age) of a node 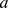 by 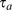.

#### Extension of the Wilson-Balding operator

We extend the Wilson-Balding operator (a type of subtree prune and regraft) [40] to sampled ancestor trees so that it is identical to the original implementation in BEAST [41] when it is restricted to trees with no sampled ancestors. The operator may propose a significant change to a tree and may change its dimension, that is, the number of nodes in the tree. We use the reversible jump formalism of [39].

First, we describe a reduced version of the operator that does not change the root. Let 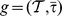 be a genealogy. There are three steps in proposing a new tree.

1. Choose edge 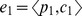 uniformly at random such that 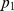 is not the root (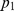 is the parent of 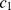). Recall that we do not consider the origin as a node belonging to the tree.
2. Choose either edge 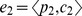 or leaf 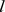. The method of selection depends on the type of 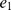:
  - if node 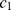 has a sibling then, uniformly at random from all possibilities, either choose edge 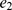 which is not adjacent to 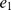 and at least one end of which is above 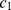 (i.e., 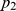 is older than 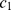) or leaf 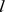 which is older than 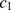;
  - if node 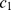 does not have a sibling (so 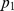 has only one child, i.e., it has degree two and thus is a sampled node) then choose edge 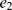 such that at least one of its ends is older than 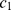 or a leaf which is older than 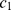 uniformly at random.

If there is no such edge nor leaf then the proposal is rejected.

3. If an item was chosen in step 2, then prune the subtree rooted at node 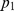 and reattach it to edge 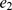 or leaf 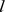. When attaching to an edge, we draw a new height for the parent of node 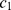 uniformly at random from the interval 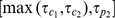.

**Figure 2:**
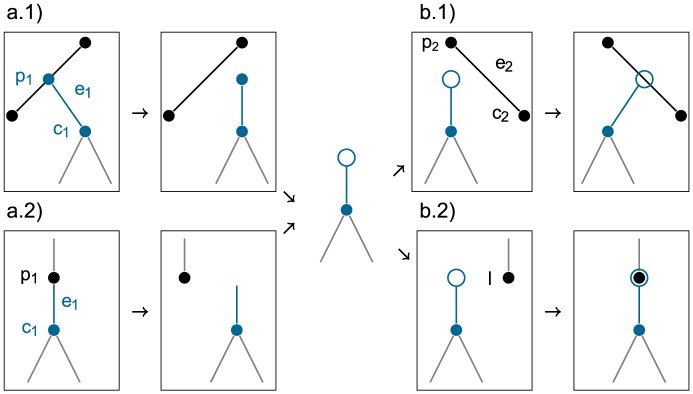
The Wilson Balding operator. The operator proposes a sampled ancestor tree topology and node ages and may propose a tree of larger or smaller dimension (the number of nodes in the tree) than the original tree. First, it prunes a subtree rooted at edge 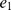 (blue edge) either from a branch, coloured black, in case a.1 or from a node, coloured black, in case a.2. Then it attaches the subtree either to an edge 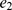 (black edge) at a random height in case b.1 or to a leaf 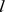 (black node) in case b.2. Case a.1 followed by b.2 removes a node from the tree and case a.2 followed by b.1 introduces a new node into the tree.

Figure 2 illustrates pruning from a branch (case 2a) and from a node (case 2b) and attaching to a branch and to a leaf. Let the resulting new genealogy be 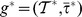.

Now we extend this move to add the possibility of changing the root. We modify the described procedure as follows. We allow 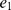 for which 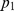 is the root to be chosen at the first step, and we allow the root edge (i.e., the edge which connects the root with the origin) to be chosen at the second step. Although we do not usually consider this edge as a part of the tree, for convenience we assume we can choose it. In this case, the parent of node 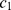 becomes a new root with the height obtained by drawing a difference between the new root height and the old root height from the exponential distribution with rate 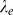.

To calculate the Hastings ratio, 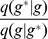, for this move we derive the proposal density, 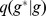. 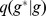 is a product of the probability of choosing edge 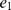 at the first step, the probability of choosing edge 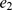 (or leaf 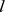) at the second step, and the probability density of choosing a new age at the third stage (or one if we attach to a leaf).

Let 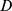 denote the number of edges in tree 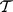. Then the contribution of the first step to the proposal density is 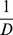. The probability at the second step depends on the number of choices there. However, since we choose the same subtree to prune in the forward and backward moves and then, at step two, choose from the items remaining in the tree after pruning the subtree, the second terms in the product will cancel in the ratio and we do not calculate them.

The contribution of the third step depends on the type of move. When attaching to a leaf it is equal to one. When attaching to a branch it is equal to the probability density of a random variable 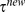 which defines a new age for the parent of 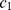. So it is either 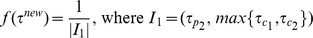
or 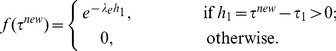
where 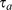 denotes the height of node 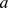. The Hastings ratio for the different cases is summarised in Table 1.

**Table 1:** Hastings ratio for the extension of the Wilson Balding operator.

Pruning from/Attaching to	internal branch	leaf	root branch
internal branch	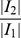	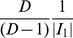	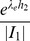
internal node	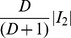	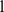	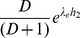
root branch	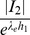	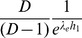	-

The table summarises the Hastings ratio 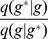 for the extended Wilson Balding operator.

#### Leaf to sampled ancestor jump

This is a dimension changing move that jumps between two trees where a particular sampled node is a sampled ancestor in one tree and a leaf in the other. The proposal starts by randomly choosing a sampled node 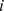. If 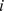 is a sampled ancestor, we propose a new tree where 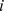 is a leaf as follows. Let 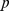 be the parent of 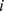 and 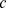 be the child of 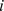. Create a new node 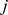 with height chosen uniformly at random from the interval 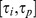. Make 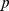 the parent of 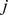 and make 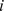 (now a leaf) and 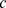 the children of 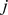.

If 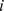 is a leaf then it becomes a sampled ancestor replacing its parent if possible. It is not possible if 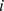 has no sibling or the sibling of 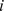 is older than 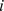. When this is possible, let node 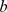 be the parent of 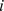 in the proposed tree. The Hastings ratio for this move is 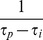 when 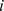 is a sampled ancestor and 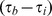 when 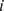 is a leaf.

Note that these same trees can be proposed under the extended Wilson-Balding operator. We introduce this more specific, or local, operator to improve mixing.

#### Other operators

We extend the narrow and wide exchange operators used in BEAST2 [6] to sampled ancestor trees. The narrow exchange operator swaps a randomly chosen node with its aunt if possible. It chooses a non-root node 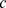 such that its parent 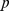 is not the root either. If the parent 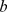 of node 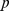 is not a sampled node and, therefore, has another child 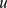 and the height of 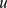 is less than the height of 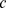 then we remove edges 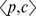 and 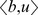 and add edges 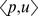 and 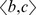. Otherwise the proposal is rejected. The wide exchange operator swaps two randomly chosen nodes along with the subtrees descendant from these nodes if none of them is a parent to another one and the ages of the parents allow to swap the children. The Hastings ratio is 1 for both operators.

To propose height changes we use a scale operator and a uniform operator. The scale operator scales non-sampled internal nodes by a scale factor drawn from the uniform distribution on the interval 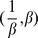, where 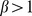. If the scaling makes some parent node younger than either of its children then the proposal is rejected. The Hastings ratio for this operator is 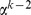, where 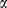 is the scale factor and 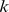 is the number of internal non-sampled nodes (the number of scaled dimensions). The uniform operator proposes a new height for internal nodes chosen uniformly at random from the interval bounded by the heights of the parent and the oldest child of the chosen node. The Hastings ratio for this operator is 1.

### Simulations and empirical data analysis

#### Simulating the fossilized birth-death process

We simulated 100 trees under the fossilized birth-death model (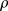-sampling and 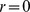). We fix the tree model parameters in this simulation:
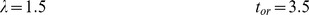

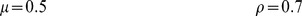

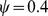
Since the time of the origin is one of the model parameters, we simulate each tree on the time interval of 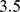. We discard trees with less than five sampled nodes, which constitute 8% of the simulated trees. The remaining trees have 55 sampled nodes on average. Then we simulated sequences along each tree under the GTR model with a strict molecular clock model and ran the MCMC with the sequences and sampled node dates as the input data. Note that the simulated data includes sequences for 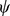-sampled nodes. For these runs, we use the re-parameterisation: 
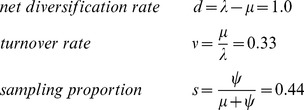
(8)along with the time of origin, 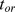 and 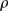. The sampling proportion is the proportion of individuals which are sampled before they are removed, meaning it is the proportion of sampled individuals out of all individuals in the full tree. In this parameterisation there are only two parameters (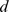 and 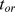) on the unbounded interval 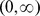 with the others are defined on 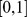, making it a convenient parametrisation for defining uninformative priors. For the tree prior distribution we use the distribution with probability density function (2) multiplied by priors for hyper parameters: 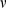, 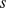, and 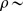 Uniform(0,1) for and Uniform(0,1000) for 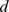 and 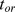.

We estimate a tree, macroevolutionary parameters, GTR rates, and the clock rate. The parameters of interest include the macroevolutionary parameters (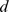, 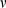, 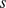, and 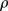) and features of the tree including the time of the origin (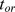), tree height and the number of sampled ancestors.

Further, we use the same simulated data to investigate the inferential power of the fossilised birth-death model in the absence of molecular data for 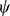-sampled nodes (e.g. to represent fossil samples in real data sets). We ran the MCMC with sequence data from contemporaneously sampled nodes and only sampling dates (but not sequences) for the 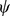-sampled nodes. Since the input data does not contain the topological locations of fossil nodes, we also need to fix one of the parameters to the truth. We chose to fix sampling probability, 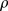, because it is likely to be known in analyses of real datasets. Note that we sample full genealogies, which include both extant and fossil samples. It is impossible to estimate the topological position of the fossil nodes without sequence or morphological data but sampling full genealogies accounts for this uncertainty.

#### Simulating the transmission birth-death process

In our second set of simulations, there is no 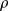-sampling but 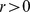. Here we again use 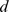, 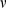, and 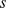 parametrisation defined by Equations (8). We fix the time of the origin, 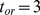, and draw the tree model parameters from the distributions 
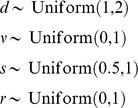
and simulate a tree under the transmission birth-death process with drawn parameters on the fixed time interval. We choose these prior distributions because they cover a wide range of parameter combinations of interest and produce trees of reasonable size. We discard trees with less than 5 or greater than 250 sampled nodes, which constitute 21% of the sample. In total, we report the results on 100 trees with the mean number of sampled nodes being 53. We simulate sequences along each tree under the GTR model with a strict molecular clock.

In the MCMC runs, we fix the sampling proportion, 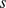, to its true value as only three out of the four transmission birth-death parameters can be inferred. We chose to fix 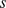 because it is one of the parameters about which there is likely strong prior knowledge in a typical epidemiological study. The tree prior distribution is (1) with uniform prior distributions for hyper parameters 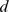, 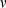, 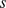, and 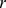, on the same intervals as above and Uniform(0,1000) prior distribution for the time of the origin. We estimate the tree, tree model parameters, GTR rates and clock rate and assess the estimates of the tree model parameters and properties of the tree.

To assess the bias introduced by model misspecification we also analyse these simulated datasets under the tree prior model without sampled ancestors, that is, we fix the removal probability 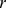 to one for the inference. Fixing 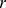 to one results in any tree with sampled ancestors having probability density zero. Thus any proposed tree with sampled ancestors is rejected in the MCMC which is equivalent to not allowing sampled ancestor trees.

#### Simulating under the sampled ancestor skyline model

We simulated the skyline transmission process under three different sets of parameters and then estimated the parameters in MCMC with fixed trees and with some parameters fixed. We have tried scenarios with two and three intervals, fixing either 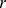 or 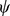. In one scenario, only 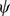 changes through time from zero to a non-zero value and the other parameters stay constant. In the second scenario, all parameters except 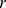 change through time. In the final scenario, all parameters change through time and the whole vector 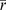 is fixed in the inference. For a full description of the parameter and prior settings see Text S1.

#### Bear dataset analysis

We re-analyzed the bear dataset from [30] comprised of sequence data of 10 extant species and occurrence dates of 24 fossil samples, assigned to six clades. Heath *et al.* [30] assume that the tree topology on the extant species is known and each fossil sample is assigned to a clade in the tree, i.e., each fossil sample is constrained to be a descendant of a particular node in the extant tree. Here, we replicate this analysis using the MCMC implementation of the fossilized birth-death model in BEAST2.

The fossilized birth-death model we use is the same model as in the original analysis by Heath *et al.* [30] but we use a strict clock instead of a relaxed molecular clock model. We perform two analyses, both with a strict clock, using our implementation in BEAST2 and the implementation in DPPDiv by Heath *et al*.

The tree prior density is (3) with transformed parameters 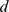, 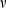, and 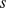 for which we chose uniform priors and 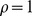 is fixed. We use the strict molecular clock with an exponential prior for the clock rate and the GTR model with gamma categories with uniform priors for GTR rates and gamma shape.

The prior distributions in both analyses (in BEAST2 and DPPDiv) are all the same except the priors for GTR rates and gamma shape. In DPPDiv, 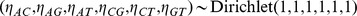
In BEAST2, we fix 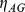 to one and use Uniform(0, 100) priors for other rates. We used a uniform prior for the gamma shape parameter in BEAST2 and an exponential prior in DPPDiv.

#### HIV 1 dataset analysis

We re-analyzed UK HIV-1 subtype B data from [42] consisting of viral sequences obtained from 62 patients (one sequence per patient). We use the skyline model without 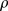-sampling and with one rate shift time (in 1999) because no samples were taken before this time. The tree prior density is (5). We use the following parameterisation and prior distributions: 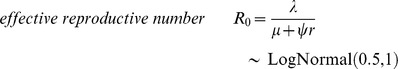



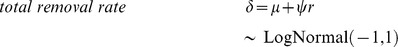

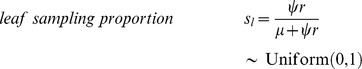

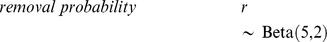

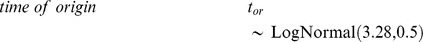
The leaf sampling proportion is the proportion of individuals who are removed by sampling out of all removed individuals, thus it is the proportion of sampled tips out of all tips in the full tree. The parameterisation and prior distributions are different from the distributions used in simulation studies. We chose the prior distributions for 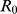, 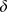, and 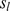 following [23] and the prior distribution for 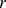 assuming that diagnosed patients are likely to change their behaviour. Recall that this model is unidentifiable and we need to have a good prior knowledge about at least one of the parameters.

We suppose that only leaf sampling proportion changes through time and it changes from zero to a non-zero value in year 1999. Other parameters stay constant through time. We use a GTR model with gamma categories and a molecular clock model with the substitution rate fixed to 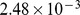 as was estimated in [23].

## Results

We developed a Bayesian MCMC framework for phylogenetic inference with models that allow sampled ancestors. We implemented a sampled ancestor MCMC algorithm as an add-on to software package BEAST2 [6] thereby making several sampled ancestor birth-death prior models available to users. We test the accuracy and limitations of these models in simulation studies and apply the sampler to infer divergence times for a biological dataset comprised of extant species and fossil samples and to an HIV dataset. In the case of the fossil-bear dataset, we compare the results obtained from our implementation to the result obtained from an alternative implementation [30].

### Simulation of sampled ancestor models

We simulated the sampled ancestor birth-death process and sampled ancestor skyline process under different scenarios. In all cases, the simulations show that we can recover the tree and model parameters from sequence data and sampling times. In the analyses where sampled ancestors were not accounted for, the estimates of the tree branching model parameters and clock rate were biased. The bias and low accuracy were the most pronounced for the birth rate (or diversification rate in the alternative parameterisation).

For some variants of the model, one of the tree model parameters has to be fixed for the inference to its true value as was discussed in the Methods section. Simulation studies show that fixing one of the parameters allows the recovery of the remaining parameter values. In particular, we showed that function (1) depends exactly on three parameters because fixing 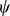 allows recovery of 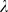, 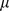 and 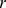 while function (2) depends on all four parameters: 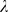, 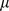, 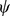 and 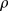. We also simulated scenarios where we fixed different parameters, for example, 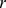 or 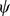. All scenarios give accurate estimates of the remaining parameter values.

We present here detailed results of two sets of simulations: one for the fossilized birth-death process and another one for the transmission birth-death process. Further simulation results can be found in the Supporting Information (Tables 2–6 in Text S1).

In these two scenarios, we first simulated trees and then sequences along the trees. Then we ran the sampler to recover tree model parameters and genealogies from simulated data comprised of sequences and sampling times. For the simulated fossilised birth-death process, we also performed analyses where only extant sequences are used. In this case, we still estimate full topologies that include fossil and extant samples to account for the uncertainty in topological locations of the fossil samples. We assess the results by calculating summary statistics including: the median estimate of a parameter, the relative error and relative bias of the median estimate, and the relative width of the 95% highest posterior density (HPD) interval. We assess whether the true value belongs to the 95% HPD interval. To summarise the results from a collection of runs we calculate the medians of the summary statistics (i.e, the median of the estimated medians, the median of the relative errors and so forth) and count the number of times when the true value belongs to the 95% HPD interval [43]. To assess the power of the method with regard to estimation of sampled ancestors we performed the receiver operating characteristic analysis [44] which estimates false positive and false negative error rates under different decision rules.

**Figure 3:**
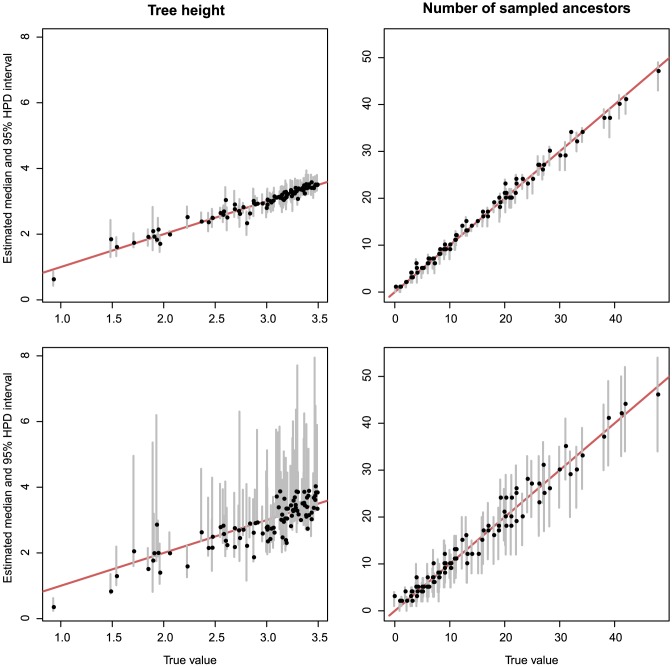
Properties of the tree estimated from simulated data (fossilized birth-death process). The graph shows median estimates (black dots) and 95% HPD intervals (grey lines) against true values for the tree height (on the left) and number of sampled ancestors (on the right). The upper row shows the estimates obtained from the analyses of simulated sequence data of all sampled nodes and the bottom row shows the estimates from the analyses where only sequence data from the extant samples was used.

**Figure 4:**
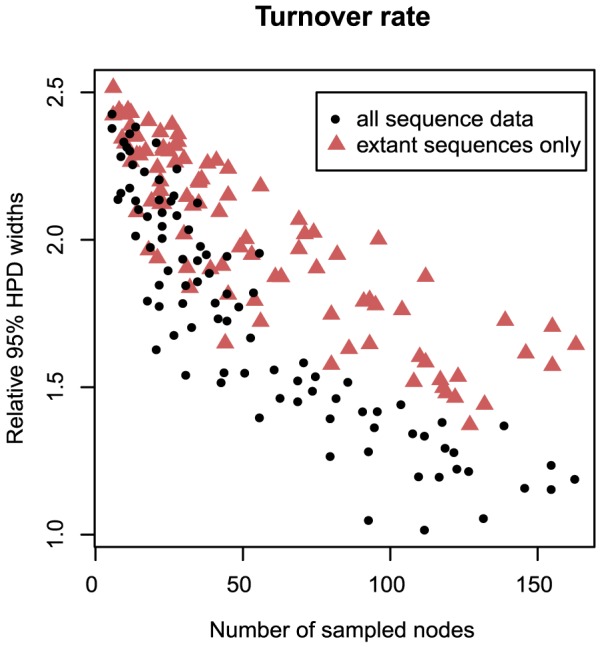
Uncertainty in estimates for simulated data (fossilized birth-death process). The graph shows the widths of relative 95% HPD intervals of the turnover rate, 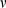, against tree sizes for simulated fossilized birth-death process. The black dots are the interval widths for posterior distributions obtained from the analyses of simulated sequence data of all sampled nodes and the red triangles are the interval widths from the analyses of sequence data of only extant samples.

For the fossilized birth-death process (the process with 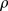-sampling and zero removal probability), we simulated a set of trees under a fixed set of the tree model parameters. In the case when we analysed sequence data of all sampled nodes, each parameter was estimated and, in the worst case, the median of the relative errors for all runs was 0.22 (0.24 for the analyses without 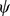-sampled sequences). The median of the relative errors for tree properties, such as the time of origin, tree height and number of sampled ancestors, was at most 0.09 (0.14 without 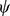-sampled sequences). The true parameters and tree properties were within the estimated 95% HPD intervals at least 95% (93% without 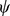-sampled sequences) of the time in all cases. The estimates of the number of sampled ancestors and the tree height for both cases are shown in Figure 3. Figure 4 shows how the amount of uncertainty in estimates of turnover rate decreases with the size of the tree (i.e., with the number of sampled nodes) and increases when the sequences of 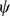-sampled nodes are discarded. Overall removing sequence data of 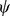-sampled nodes led to larger errors and increased 95% intervals. The median of errors for the turnover rate and sampling proportion were comparable as was the coverage for all macroevolutionary parameters. This might be due to fixing 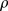 to the truth. The detailed results of this set of simulations can be found in Supporting Information (Table 4 in Text S1).

**Figure 5:**
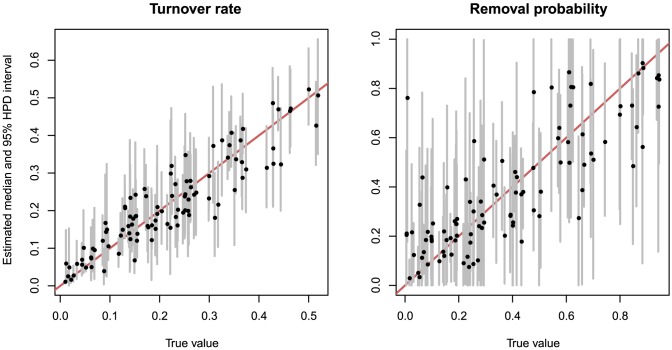
Parameter estimates for simulated data (transmission process). The graph shows median estimates (black dots) and 95% HPD intervals (grey lines) against true values for the turnover rate, 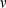, (on the left) and removal probability, 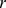, (on the right).

To simulate from the transmission birth-death process, i.e., the sampled ancestor birth-death process without 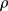-sampling and with non-zero removal probability, we draw tree model parameters from uniform distributions for each simulation. The tree model parameters were estimated with a maximum median of relative errors of 0.28 and, for the tree properties, of 0.06. In the worst case a parameter or a tree property was inside the 95% HPD interval 92% of the time. The estimates of the parameters are shown in Figure 5. When sampled ancestors were not accounted for the time of origin was accurately estimated but the tree height and model parameters were substantially biased. The median of the relative biases of the tree height increased from 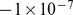 to 0.01, for the diversification rate from 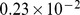 to 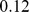 (Figure 1 in Text S1). When sampled ancestors were not accounted for in the inference the true tree height was inside 95% HPD interval 82% of the time, diversification rate 69%, and turnover rate 85%. More detailed results are presented in Table 5 in Text S1.

**Figure 6:**
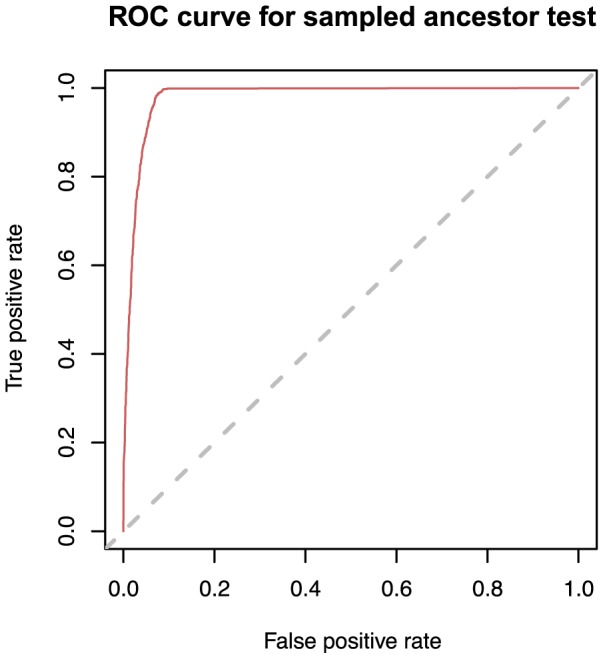
ROC curve for identifying sampled ancestors based on simulated data (transmission process). The posterior distribution of trees obtained from a Bayesian MCMC analysis of simulated sequence data can be used to detect sampled ancestors. We identify a node as being a sampled ancestor if the posterior probability that the node is a sampled ancestor is greater than some threshold. The curve is parameterised by the threshold and shows the trade-off between true positive rate (sensitivity) and false positive rate (specificity) for different values of the threshold (any increase in sensitivity will be accompanied by a decrease in specificity). The dashed diagonal line corresponds to a ‘random guess’ test. The closer the ROC curve to the upper-left boarder of the ROC space (the whole area of the graph), the more accurate the test. The optimal value of the threshold for this curve is 0.45.

We used the data simulated from the transmission process to perform the receiver operating characteristic (ROC) analysis of the sampled ancestor predictor, which makes a prediction relying on the posterior distribution of genealogies. A node is predicted to be a sampled ancestor with a probability calculated as a fraction of trees in the posterior sample in which the node is a sampled ancestor. Out of the 5225 total sampled nodes in all simulated trees (excluding the last sample in each tree because this cannot be a sampled ancestor), 1814 were sampled ancestors. The ROC curve constructed from this data and predictions obtained from the MCMC runs is shown in Figure 6.

### Application of the fossilized birth-death model to a bear dataset

**Figure 7:**
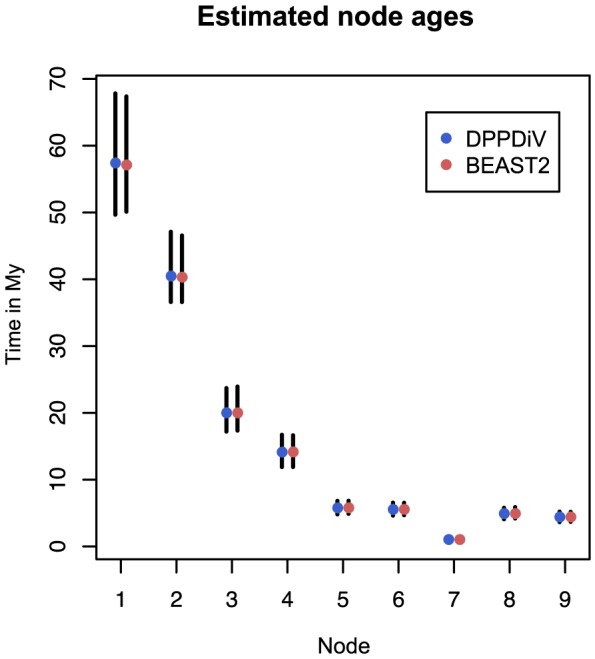
Divergence time estimates for the bear dataset. The estimates are obtained from the analyses with DPPDiv [30] (left bars with blue dots) and BEAST2 (right bars with red dots) implementations of the fossilised birth-death model, which give the same results. The bars are 95% HPD intervals and the dots are mean estimates. The node numbering follows the original analysis [30]: nodes 1 and 2 represent the most recent common ancestors of the bear clade and two outgroups (gray wolf and spotted seal). Node 3 is the most recent common ancestor of all living bear species and nodes 4-9 are the divergence times within the bear clade.

We ran two analyses of the bear dataset originally analysed in [30] with BEAST2 and with the DPPDiv implementation by Heath *et al.* under the same model. The tree topology relating all living bear species and two outgroup species is fixed in the analyses and we estimate the divergence times and three tree model parameters: 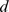, 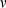, and 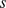 since the sampling probability 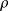 was fixed to one in the inference. The estimates are the same in both analyses as expected. The estimated divergence times are shown in Figure 7. The median estimate and 95% HPD interval for the net diversification rate, 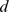, were 0.027 per million years and [0.002, 0.058]; for the turnover rate, 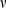, 0.51 and [0.1, 0.9]; and for the sampling proportion, 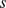, 0.77 and [0.46, 0.98]. Most of the fossil samples were estimated to be direct ancestors of extant species or other fossil species, that is, the median estimate of the number of sampled ancestors was 22 with 95% HPD interval [17], [24].

### Application of sampled ancestor skyline model to HIV dataset

We analysed an HIV-1 subtype B dataset from the United Kingdom, consisting of 62 sequences that were originally analysed in [42] and later analysed using the skyline model without sampled ancestors in [23]. For three of the sampled nodes the posterior probability of being a sampled ancestor was 61%, 59%, and 49%, respectively. For all other sampled nodes the posterior probability was less than 4%. There is positive evidence that the three sampled nodes with high posterior probabilities are sampled ancestors. The Bayes factors are 5.9, 8.7, and 4.2, respectively.

**Figure 8:**
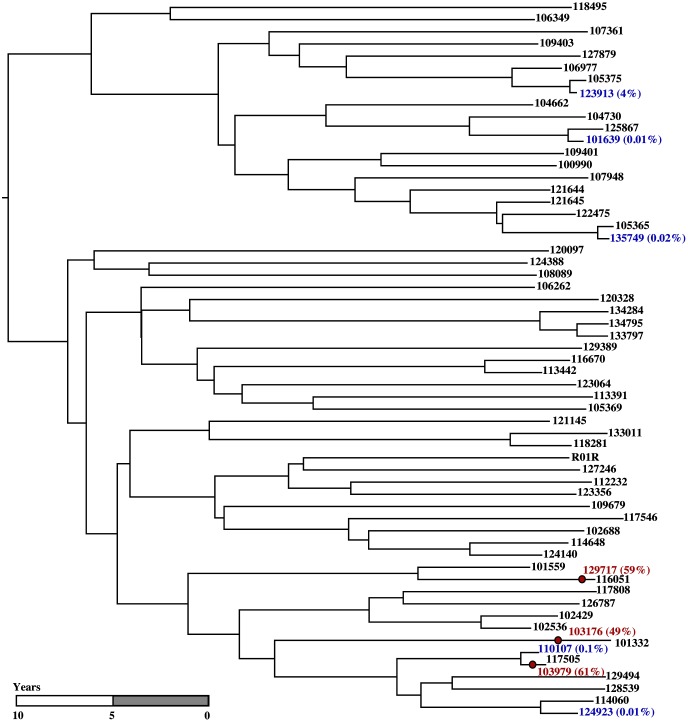
A tree sampled from the posterior of the HIV 1 dataset analysis. The tree exhibits three estimated sampled ancestors shown as red circles. The samples with positive posterior probabilities of being sampled ancestors are shown in colour (red for the nodes with evidence of being sampled ancestors and blue for other nodes with non-zero probabilities) with the posterior probabilities in round brackets.

We chose a random tree among the trees in the posterior sample that have exactly these three nodes as sampled ancestors. The tree is shown in Figure 8. All three sampled ancestors are clustered within a clade of 16 (out of 62) samples, suggesting that this clade was more extensively sampled. The median of the posterior distribution of the number of sampled ancestors was 2 with 95% HPD interval 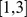. The removal probability was estimated to be 0.74 with 95% HPD interval 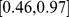, indicating a substantial reduction in the probability that infected patients remained able to cause further infections after they were diagnosed.

## Discussion

The MCMC sampler developed here enables analyses under models in which the probability of one sample being the direct ancestor of another sample is not negligible. These models are useful for describing infectious transmission processes, including identifying transmission chains. They are also useful for estimating divergence times for macroevolutionary data in the presence of fossil samples.

In the analysis of a phylogeny of bears we show that the sampler can be applied to data comprised of both fossil and recent taxa to infer divergence times. This dataset was previously analysed using the *fossilized birth-death model* by Heath *et al.* [30]. While the underlying model is the same and thus produces the same results, there is a conceptual difference between the two MCMC frameworks. In the analysis by Heath *et al.*, MCMC was used to integrate over fossil attachment times while the topological attachment of the fossils was integrated out analytically. To achieve this, the topology of the phylogeny relating the extant taxa had to be assumed to be known. In our implementation, we average over the trees relating fossil and extant taxa, i.e., over both the fossil attachment times and topological attachment points, using MCMC. To facilitate a direct comparison we constrained the topology of the extant species, however, our implementation does not require this. For datasets where the tree topology is well resolved, analytical calculation results in faster mixing but when there is uncertainty in the extant phylogeny, which is the more common case, our sampler can account for it. Since the two implementations of the method were made completely independently of one another, this result also provides strong evidence that both implementations are sampling from the correct posterior distribution.

A natural extension to the analysis of the bear phylogeny would be to include morphological data to inform the inference regarding the precise placement of fossils on the tree [33], [34], however this requires probabilistic models of morphological character evolution [29], [45]. Another direction for application of the sampler is using the skyline version of the fossilized birth-death model to analyse datasets where fossil samples come from different stratigraphic layers, so that rates of fossilisation and discovery may change through time. Fossils are better preserved in some layers than in other layers and therefore the sampling rate varies from layer to layer (see, for example, [46]) and this can be modelled as a skyline plot.

Simulation studies show that the MCMC sampler for sampled ancestor trees allows for the detection of direct ancestors within the sample given sequence data and sampling dates. The simulation scenario where sequences were removed from the fossil samples demonstrates that the tree model is informative about sampled ancestors given that the sequence data from contemporaneous samples, sampling dates of fossils and sampling probability, 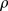, are known.

The posterior probability that a sample is a sampled ancestor is comprised of two components. For the simple two sequence case, one component is the probability that the amount of difference observed in two sequences with time 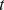 between sampling is a result of the underlying substitution process that lasted for a period of time close to 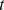. The second component is the prior probability, 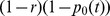, that the earlier sample is a sampled ancestor. The two probabilities depend on the substitution rate and tree model parameters, respectively, that are jointly estimated. We have shown that these parameters, and therefore which samples are sampled ancestors, can be accurately inferred given sufficiently many and sufficiently long sequences and sampling dates.

In epidemiological studies, sampled ancestors can be interpreted as sampled individuals that have later infected other individuals. In the analysis of the HIV dataset, we equated the transmission tree directly with the viral gene tree. This approximation is good enough to demonstrate the method. But for chronic infectious diseases such as Hepatitis C and HIV where the genetic diversity of the pathogen population within a single host can be substantial (e.g. [20], [47]) the inferential power would be improved by a hierarchical model that explicitly models the difference between the sampled ancestor transmission tree and the (binary) viral gene tree. Regardless of the modelling details, such analyses allow for the estimation of the removal at sampling parameter 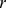, which controls the prevalence of sampled ancestors. In most situations this parameter reflects the probability with which patients remain able to cause further infections after they were diagnosed.

Even if the sampled ancestors are not of specific interest in an analysis it is important to model sampled ancestors when the data is likely to contain them because failing to do so introduces a bias to the estimates of the parameters. The birth rate, diversification rate and clock rate were all substantially biased when sampled ancestors were not accounted for.

Analytic calculations (presented in Text S1) and simulation studies show that there is a degree of non-identifiably of parameters in the transmission birth-death models that include the 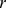 parameter. In other words, these models require one of the parameters to be fixed or strongly constrained by prior information to achieve unambiguous inference. In epidemiological studies with a known sampling scheme, a candidate parameter to fix is the sampling proportion. For epidemics with a well-characterised period of infection, such as influenza, the total removal rate, 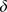, could be fixed. Under the fossilized birth-death model, it is possible to infer all the parameters of the tree process prior when time-stamped comparative data is available. This is an interesting insight: if no fossils are available, we can only infer two out of the three parameters 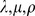 (as the likelihood only depends on 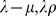) while in presence of fossils we can estimate all four parameters 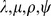 (as the likelihood depends on 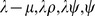).

The fossilised birth-death model allows the inference of tree model parameters given the phylogeny or time-stamped comparative data. The simulation study showed that without comparative data for fossil samples and assuming the sampling probability, 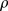, is known, it is still possible to infer the tree model parameters and phylogenies (excluding the phylogenetic positions of the fossil nodes) albeit with increased uncertainty. In the bear data analysis, we used this type of input data (extant sequences, fossil occurrence dates and fixed 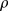) and additionally imposing monophyletic constraints on the fossils. Including comparative data for the fossil samples would have allowed inference about their precise phylogenetic placement without imposing monophyletic constraints. As sequence data for fossil organisms is rarely available information about fossil locations on the tree obtained by phylogenetic modelling of morphological data [29], [45] may become important to enable effective inference. This approach has been termed *total evidence fossil dating* [29] and is the subject of active research.

The implementation of the sampled ancestor skyline model assumes that the rate shift times are known *a priori*. However, there are methods that relax this assumption for the skyline model without sampled ancestors. In one such method, the change-points are considered to be equidistant and only the number of the intervening intervals needs to be known prior to the inference [23]. Another method infers both the rate shift times and the number of shifts [48]. Similar methods are yet to be developed for the skyline model with sampled ancestors. The identifiability of parameters (including or excluding times of the rate shifts) of the skyline model also remains to be investigated.

To our knowledge this is the first full implementation of an MCMC sampler for sampled ancestor trees and we anticipate that such samplers will form the computational basis for further developments in fossil-calibrated divergence time dating, total-evidence fossil dating and phylodynamics.

## Supporting Information

Text S1 **Supporting information.** The text describes equation derivations, details of simulation studies presented in the main text and additional simulation studies, and other supporting information.
(PDF)

File S1 **Sampled ancestor package setup instructions.**
(TXT)

File S2 **XML file for the bear dataset analysis.**
(XML)

File S3 **XML file for the HIV-1 dataset analysis.**
(XML)

File S4 **XML files for simulated fossilized birth-death process.**
(ZIP)

File S5 **XML files for simulated transmission process.**
(ZIP)

File S6**XML files for simulated fossilized birth-death process without **
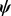
**-sampled sequences.**(ZIP)

File S7**XML files for simulated transmission process with **
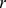
** fixed to one.**(ZIP)
