# Supplementary material for: Bayesian Inference of Sampled Ancestor Trees for Epidemiology and Fossil Calibration
Source: PLoS Comput Biol. 2014 Dec 4;10(12):e1003919. doi: 10.1371/journal.pcbi.1003919 (PMC4263412; doi:10.1371/journal.pcbi.1003919)
Supplement: Text S1 — Supporting information. The text describes equation derivations, details of simulation studies presented in the main text and additional simulation studies, and other supporting information. (PDF) [file pcbi.1003919.s001.pdf]

Bayesian inference of sampled ancestor trees for epidemiology and fossil calibration

Supporting Information

Sampled ancestor skyline model

Theorem 1. *The probability density function for a reconstructed tree $g = (\mathcal{T}, \bar{x}, \bar{y}, \bar{z})$ produced by the sampled ancestor birth-death skyline process with parameters $\bar{\lambda}, \bar{\mu}, \bar{\psi}, \bar{r}, \bar{\rho}, \bar{t}$ is equal to*

$$\begin{aligned} f[g|\bar{\lambda}, \bar{\mu}, \bar{\psi}, \bar{r}, \bar{\rho}, \bar{t}] = &\frac{2^{m+M-1}}{(m+M+k+K)!}\times \\ &q_1(t_0)\prod_{i=1}^{k}(1-r_{\mathbf{i}_{z_i}})\psi_{\mathbf{i}_{z_i}}\prod_{i=1}^{m+M-1}\lambda_{\mathbf{i}_{x_i}}q_{\mathbf{i}_{x_i}}(x_i)\prod_{i=1}^{m}\frac{\psi_{\mathbf{i}_{y_i}}(r_{\mathbf{i}_{y_i}}+(1-r_{\mathbf{i}_{y_i}})p_{\mathbf{i}_{y_i}}(y_i))}{q_{\mathbf{i}_{y_i}}(y_i)}\times \\ &\prod_{i=1}^{l}((1-\rho_i)q_{i+1}(t_i))^{n_i}\rho_i^{N_i}((1-r_{i+1})q_{i+1}(t_i))^{K_i}(r_{i+1}+(1-r_{i+1})p_{i+1}(t_i))^{M_i}, \end{aligned} \tag{1}$$

where, for $i = 1, \ldots, l$ and $t_i \le t < t_{i-1}$,

$$p_i(t) = \frac{\lambda_i + \mu_i + \psi_i - A_i\frac{e^{A_i(t-t_i)}(1+B_i)-(1-B_i)}{e^{A_i(t-t_i)}(1+B_i)+(1-B_i)}}{2\lambda_i}$$

with

$$A_i = \sqrt{(\lambda_i - \mu_i - \psi_i)^2 + 4\lambda_i\psi_i}$$

and

$$B_i = \frac{(1-2(1-\rho_i)p_{i+1}(t_i))\lambda_i + \mu_i + \psi_i}{A_i};$$

$p_{l+1}(t_l) = 1$*; and, for $i = 1, \ldots, l$,*

$$q_i(t) = \frac{4e^{A_i(t-t_i)}}{(e^{A_i(t-t_i)}(1+B_i)+(1-B_i))^2}.$$

The other notation is summarised in Table 1

Proof. First, we consider the same process but where at each bifurcation time, we label one of the new lineages as *left* and another as *right* and we do not label sampled nodes. In this case, the process produces oriented trees instead of labeled trees.

Table 1: **Sampled ancestor Skyline model notation**

Notation	Description
l	the number of intervals or parameter shift times,
t_{or}	$t_{or} = t_0$, the time of origin,
t_i	a parameter shift time or ρ-sampling time for $i \in \{1, \ldots, l\}$ with $t_l = 0$,
$\bar{t}$	$(t_0, \ldots, t_{l-1})$ is a vector of time parameters that are necessary to define the model,
m	the number of ψ-sampled tips,
$\bar{y}$	$(y_1, \ldots, y_m)$ is a vector of times of ψ-sampled tips,
M_i	the number of tips sampled at time t_i for $i \in \{1, \ldots, l\}$,
M	$\sum_{i=1}^{l} M_i$,
$\bar{x}$	$(x_1, \ldots, x_{m+M})$ is a vector of bifurcation times,
k	the number of ψ-sampled nodes that have sampled descendants,
$\bar{z}$	$(z_1, \ldots, z_k)$ is a vector of times of ψ-sampled nodes with sampled descendants,
K_i	the number of nodes, with sampled descendants, sampled at time t_i for $i \in \{1, \ldots, m\}$
K	$\sum_{i=1}^{l} K_i$,
N_i	$K_i + M_i$ the total number of nodes sampled at time t_i for $i \in \{1, \ldots, l\}$,
n_i	the number of lineages presented in the tree at time t_i but not sampled at this time for $i \in \{1, \ldots, l\}$,
$\mathbf{i}_x$	an index such that $t_{\mathbf{i}_x} \leq x < t_{\mathbf{i}_x - 1}$.

The probability $p_i(t)$ that an individual alive at time t has no sampled descendants when the process is stopped (i.e., in the time interval $[t_l, t]$), with $t_i \leq t < t_{i-1}$ ($i = 1, \dots, l$) was derived in [23] for the birth-death skyline model without sampled ancestor (i.e., $r = 1$).

Consider an event that the individual that started the process at time t_{or} has no sampled descendants in the time interval $[t_l, t_{or}]$. This event does not depend on the behaviour of the process in case when an individual was sampled (such as the possibility to remain in the process after sampling if $r < 1$) because it states that no individual was sampled. That implies $p_1(t_{or}|\lambda, \mu, \psi, r) = p_1(t_{or}|\lambda, \mu, \psi)$. Since the evolution of each lineage is independent of the evolution of other coexisting lineages under this model, we can say the same about the event that an individual alive at some time $t < t_{or}$ has no sampled descendant when the process is stopped, that is, $p_i(t|\lambda, \mu, \psi, r) = p_i(t|\lambda, \mu, \psi)$ for i as above.

For convenience, we split every edge existing at time t_i (for $i = 1, \dots, l-1$) with a two degree node dated at this time. Let $g_{i,e}(t)$ be the probability density that an infected individual in the tree at time t corresponding to edge e (with $t_i \leq t \leq t_{i-1}$) evolved between t and the present as observed in the tree.

The Master equation for $g_{i,e}(t)$ along an edge with starting time t_s and ending time t_b ($t_e \leq t \leq t_b$) is

$$\frac{d}{dt} g_{i,e}(t) = -(\lambda_i + \mu_i + \psi_i) g_{i,e}(t) + 2\lambda_i p_i(t) g_{i,e}(t)$$

Note that r does not occur in the equation and will only be introduced in the initial values of $g_{i,e}$. The solution to this equation is given in [23]:

$$g_{i,e}(t) = g_{i,e}(t_e) \frac{q_i(t)}{q_i(t_e)}.$$

Further, the initial values are, for $t_e \neq t_i$,

$$g_{i,e}(t_e) = \begin{cases} \lambda_i g_{i,e_1}(t_e) g_{i,e_2}(t_e) & \text{if } e \text{ has two descendant edges } e_1,\ e_2, \\ \psi_i(r_i + (1 - r_i) p_i(t_e)) & \text{if } e \text{ is a leaf edge, and} \\ \psi_i(1 - r_i) g_{i,e_1}(t_e) & \text{if } e \text{ has one descendant edge } e_1; \end{cases}$$

and for $t_e = t_i$,

$$g_{i,e}(t_e) = \begin{cases} (1 - \rho_i) g_{i+1,e_1}(t_e) & \text{if } e \text{ has one descendant edge } e_1 \text{ and} \\ & e \text{ is not a sampled node,} \\ \rho_i(1 - r_{i+1}) g_{i+1,e_1}(t_e) & \text{if } e \text{ has one descendant edge } e_1 \text{ and} \\ & e \text{ is a sampled node, and} \\ \rho_i(r_{i+1} + (1 - r_{i+1}) p_{i+1}(t_e)) & \text{if } e \text{ is a leaf edge.} \end{cases}$$

Then the probability density of the genealogy is

$$f[g|\bar{\lambda}, \bar{\mu}, \bar{\psi}, \bar{r}, \bar{\rho}, \bar{t}] = g_{1,e_{root}}(t_0)$$

Traversing the tree from the tips to the root and deriving $g_{i,e}(t_e)$ for each time t_e (note that $q_i(t_i) = 1$), we derive that $g_{1,e_{root}}(t_0)$ is as in (1) without the first term, which comes from the fact that we considered oriented trees instead of labeled trees.

Indeed, the expression without the first term is the probability density function for oriented trees. Having the model parameters fixed (including $\bar{t}$), it only depends on branching times, sampling times of tips, sampling times of two degree nodes, the number of sampled two-degree nodes at time t_i, and the number of sampled tips at time t_i (i.e., on $\bar{x}$, $\bar{y}$, $\bar{z}$, $\bar{K}$, and $\bar{M}$), but not on how the lineages are connected, i.e., not on the particular topology. The density of an oriented and labeled genealogy which has a given oriented tree embedded is the probability density of the oriented tree divided by the $(m + M + k + K)!$ possible labelings. Ignoring the 2^{m+M-1} orientations establishes the theorem. □

Re-parameterisation

Let $\rho_1, \ldots, \rho_{l-1} = 0$ and consider the re-parameterisation, collapsing the original $4l + 1$ parameters into $4l$ parameters:

$$\begin{aligned}
d_i &= \lambda_i - \mu_i - \psi_i && \text{for } i = 1, \ldots, l \\
f_i &= \lambda_i \psi_i && \text{for } i = 1, \ldots, l \\
g_i &= (1 - r_i)\psi_i && \text{for } i = 1, \ldots, l \\
h &= \rho_l \lambda_l && \\
k_i &= \frac{\lambda_i}{\lambda_{i+1}} && \text{for } i = 1, \ldots, l - 1
\end{aligned} \tag{2}$$

We will show in the following that the tree likelihood derived in Theorem 1 conditioned on ψ-sampling at least one individual and with $\rho_1, \ldots, \rho_{l-1} = 0$ depends only on the $4l$ parameters obtained from the re-parameterisation, thus in the original parameter set of size $4l + 1$, one parameter cannot be identified from the sampled tree.

Lemma 1. *Let* $\zeta_i(t) = A_i \frac{e^{A_i(t-t_i)}(1+B_i)-(1-B_i)}{e^{A_i(t-t_i)}(1+B_i)+(1-B_i)}$. *Then*

$$
\begin{aligned}
A_i &= \sqrt{d_i^2 + 4f_i} && \text{for } i = 1, \ldots, l; \\
B_i &= \frac{k_i(d_{i+1} + \zeta_{i+1}(t_i)) - d_i}{A_{i+1}} && \text{for } i = 1, \ldots, l-1; \\
B_l &= \frac{2h - d_l}{A_l}; \\
r_i + (1-r_i)p_i(t) &= \frac{g_i}{2f_i}\Big(\frac{2f_i}{g_i} - d_i - \zeta_i(t)\Big) && \text{for } i = 1, \ldots, l; \text{ and} \\
1 - p_{i+1}(t) &= \frac{d_{i+1} + \zeta_{i+1}(t)}{2\lambda_{i+1}} && \text{for } i = 0, \ldots, l-1.
\end{aligned}
$$

Proof. We show only one case:

$$
r_i + (1-r_i)p_i(t) = r_i + (1-r_i)\frac{\lambda_i + \mu_i + \psi_i - \zeta_i(t)}{2\lambda_i} =
$$

$$
\frac{(1-r_i)}{2\lambda_i}\Big(\frac{2\lambda_i r_i}{(1-r_i)} + \lambda_i + \mu_i + \psi_i - \zeta_i(t)\Big) =
$$

$$
\frac{g_i}{2f_i}\Big(\frac{2\lambda_i}{(1-r_i)} - 2\lambda_i + \lambda_i + \mu_i + \psi_i - \zeta_i(t)\Big) = \frac{g_i}{2f_i}\Big(\frac{2f_i}{g_i} - d_i - \zeta_i(t)\Big)
$$

Other equations can be verified by substituting parameters $\bar{d}$, $\bar{f}$, h, and $\bar{k}$ with expressions given in (2).

□

Theorem 2. *When* $\rho_1, \ldots, \rho_{l-1} = 0$, *the tree density function for the sampled ancestor skyline model conditioned on* ψ*-sampling at least one individual, which is*

$$
f[g|\bar{\lambda}, \bar{\mu}, \bar{\psi}, \bar{r}, \rho_l, \bar{t}, S] \propto
$$

$$
\rho_l^{N_l} \frac{q_1(t_0)}{1-p_1(t_0)} \prod_{i=1}^{k}(1 - r_{\mathbf{i}_{z_i}})\psi_{\mathbf{i}_{z_i}} \prod_{i=1}^{m+N_l-1} 2\lambda_{\mathbf{i}_{x_i}} q_{\mathbf{i}_{x_i}}(x_i) \prod_{i=1}^{m} \frac{\psi_{\mathbf{i}_{y_i}}(r_{\mathbf{i}_{y_i}} + (1-r_{\mathbf{i}_{y_i}})p_{\mathbf{i}_{y_i}}(y_i))}{q_{\mathbf{i}_{y_i}}(y_i)} \prod_{i=1}^{l}(q_{i+1}(t_i))^{n_i}
$$

can be re-parameterised with parameters given in Equations (2).

Proof. We can write this function as follows:

$$
\rho_l^{N_l}\lambda_1 \prod_{i=1}^{m+N_l-1} \lambda_{\mathbf{i}_{x_i}} \prod_{i=1}^{m} \psi_{\mathbf{i}_{y_i}} \times
$$

$$\frac{q_1(t_0)}{\lambda_1(1-p_1(t_0))}\prod_{i=1}^{k}(1-r_{\mathbf{i}_{z_i}})\psi_{\mathbf{i}_{z_i}}\prod_{i=1}^{m+N_l-1}2q_{\mathbf{i}_{x_i}}(x_i)\prod_{i=1}^{m}\frac{(r_{\mathbf{i}_{y_i}}+(1-r_{\mathbf{i}_{y_i}})p_{\mathbf{i}_{y_i}}(y_i))}{q_{\mathbf{i}_{y_i}}(y_i)}\prod_{i=1}^{l}(q_{i+1}(t_i))^{n_i}$$

From lemma 1, it follows that $q_i(t)$, $\lambda_1(1-p_1(t_0))$, and $r_i+(1-r_i)p_i(t)$ depend only on parameters $\bar{d}$, $\bar{f}$, $\bar{g}$, h, and $\bar{k}$ and do not depend on $\bar{\lambda}$, $\bar{\mu}$, $\bar{\psi}$, $\bar{r}$, and ρ_l individually. It remains to show that

$$\rho_l^{N_l}\lambda_1\prod_{i=1}^{m+N_l-1}\lambda_{\mathbf{i}_{x_i}}\prod_{i=1}^{m}\psi_{\mathbf{i}_{y_i}}$$

also depends only on $\bar{d}$, $\bar{f}$, $\bar{g}$, h and $\bar{k}$.

Note that

$$\psi_i\lambda_j = f_i k_i \dots k_i \quad \text{for } j<i,$$
$$\rho_l\lambda_i = hk_i\dots k_l \quad \text{for } j<l,$$

and we can decompose the last term in $m+N_l$ terms in either of the two forms: $\psi_i\lambda_j$ and $\psi_l\lambda_i$. □

Setting ρ_l to zero in re-parameterisation (2), we can see that function (4) in the main text depends on $4l-1$ parameters: $\bar{d}$, $\bar{f}$, $\bar{g}$ and $k_1,\dots,k_{l-1}$, out of $4l$ parameters: $\bar{\lambda}$, $\bar{\mu}$, $\bar{\psi}$ and $\bar{r}$. Also, setting $l=1$, $\lambda_1=\lambda$, $\mu_1=\mu$, $\psi_1=\psi$, and $r_1=r$ gives us that $p_1(t)=p_0(t)$, $q_1(t)=q(t)$ and that $f[g|\lambda,\mu,\psi,r,t_0,S]$ is basically the same function as in equation (1) in the main text. That means that we can re-parameterise function (1) in the main text with $\lambda-\mu-\psi$, $\lambda\psi$ and $\psi(1-r)$.

In a similar manner, we can show that when $\bar{r}=0$, $\rho_l\neq 0$ and conditioning on sampling at least one extant individual (i.e., considering skyline fossilised birth-death process with tree probability density function as in equation (5) in the main text), function $\lambda_1(1-\hat{p}_1(t)) = \lambda_1(1-p_1(t|\bar{\psi}=0))$ depends on

$$\hat{d}_i=\lambda_i-\mu_i \quad \text{for } i=1,\dots,l;$$
$$k_i=\frac{\lambda_i}{\lambda_{i+1}} \quad \text{for } i=1,\dots,l-1; \text{ and}$$
$$h=\rho_l\lambda_l.$$

Note that $\hat{d}_i=d_i-g_i$ because $r_i=0$ implying $g_i=\psi_i$ for all i. That means we can re-parameterise function (5) in the main text with (2). But for this model, we have $3l+1$ initial parameters: $\bar{\lambda}$, $\bar{\mu}$, $\bar{\psi}$, and ρ_l; and $4l$ new parameters: $\bar{d}$, $\bar{f}$, $\bar{g}$, $k_1,\dots,k_{l-1}$ and h; implying that re-parameterisation (2) does not reduce the number of parameters in function (5) in the main text.

Testing operators

We introduced a number of operators for a random walk in the space of sampled ancestor trees and implemented the operators as a sampled ancestor add-on to the BEAST2 software. To test the implementation we ran the MCMC sampler to obtain a sample from the tree distribution defined by the sampled ancestor birth-death model [15] and compare the results with calculations made in Mathematica software.

The probability density of the tree distribution is

$$f[g|\lambda,\mu,\psi,r,t_{or}] = \frac{1}{(k+m)!} q(t_{or})(\psi(1-r))^k \prod_{i=1}^{m-1} 2\lambda q(x_i) \prod_{i=1}^{m} \frac{\psi(r+(1-r)p_0(y_i))}{q(y_i)}$$

We fix sample size $n = k + m$, sampling dates $\bar{y}$ and all the model parameters except for the time of origin t_{or} placing a uniform distribution on it. So we sample genealogies from the distribution with probability density

$$f[g,t_{or}|\lambda,\mu,\psi,r;n,\bar{y}] = f[g|\lambda,\mu,\psi,r,t_{or};n,\bar{y}]f_{or}(t_{or}) \quad (3)$$

where $f_{or}(x)$ is a probability density of the origin. We set,

$$\lambda = 2 \quad \mu = 1 \quad \psi = 0.5 \quad r = 0.9$$
$$t_{or} \sim \text{Uniform}(0,\, 1000)$$
$$n = 3 \quad y_1 = 2 \quad y_2 = 1 \quad y_3 = 0$$

We run 100 MCMC analysis to test different operators that do not change sampled nodes' times.

To assess whether the obtained tree samples are from distribution (3) with the given parameters we calculate the true marginal probabilities for all non-ranked tree topologies on three sequentially sampled individuals in Mathematica. There are eight different non-ranked tree topologies. Denote them $T_1, \ldots, T_8$. To fix string representations for the topologies we label the individual sampled at time y_1 as 1, at time y_2 as 2, and at time y_3 as 3. The true probabilities are shown in the table below:

Non-ranked tree topology	String representation	Probability in %
T_1	$((3,2),1)$	77.8327
T_2	$((3,2))1$	7.8642
T_3	$((3)2,1)$	3.8657
T_4	$(3,(2,1))$	4.3189
T_5	$((3,1),2)$	4.3189
T_6	$((3)2)1$	0.4135
T_7	$(3,(2)1)$	0.6930
T_8	$((3)1,2)$	0.6930

Further we compare the estimated marginal probabilities with the true marginal probabilities. We calculate the standard errors of the estimated probabilities for each tree topology and assess whether the estimated value is within two standard errors of the true value. To calculate a standard error given by:

$$\frac{p_{true}(1-p_{true})}{\sqrt{ESS}}$$

we need to find the number of independent samples, i.e. the effective sample size (ESS). To find the ESS we assign an integer to each of the eight topologies to obtain a sample from $\{1,\dots,8\}$ instead of a tree sample and calculate the ESS for the integer sample.

The obtained results for 100 runs are summarised in the following table:

Operators [1]	Accuracy for non-ranked tree topologies (in %)							
	T_1	T_2	T_3	T_4	T_5	T_6	T_7	T_8
WB	96	96	99	95	96	98	95	95
WB	98	95	94	92	93	96	96	98
WB and S	97	95	97	98	97	98	99	97
WB, LSJ, and Ex	92	97	97	99	98	98	95	96
WB, LSJ, Ex, and S	95	94	96	98	93	92	99	98
WB, LSJ, Ex, U, and S	94	93	91	97	93	92	94	95

[1]we use abbreviations: WB for the extension of Wilson Balding, LSJ for Leaf–sampled-ancestor jump, Ex for Exchange (assuming a combination of the narrow and wide versions), S for Scale, and U for Uniform.

Simulation studies

We divide all simulations in two groups. In one group of simulations (Scenario 1), we simulate trees and then estimate tree model parameters with the trees fixed in MCMC. In the second type of simulations (Scenario 2), we simulate trees and sequences along the simulated trees and then run MCMC with sequences and sampled node dates as the input data to estimate tree model parameters, trees, and molecular model parameters. Additionally to analysing the simulated data in Scenario 2 under the method we assume for simulations, we did two further analyses for some of the Scenario 2 simulated datasets. In subtype A, we simulate data under a model with sampled ancestors and analyse it under the same model but without sampled ancestors, i.e., fixing r to one (see Scenario A2.1.1 and A2.3). This analysis reveals the bias in parameter estimates when not accounting for sampled ancestors. In subtype B, we discard the sequence data of ψ-sampled nodes and run the analysis with only sequence data of ρ-sampled nodes and sampling times of ψ-sampled nodes (see Scenario B2.2). This analysis reveals the power to infer parameters in absence of ancestral sequences which is the case for fossils.

We simulate 100 trees in all the scenarios except for the last scenario of the skyline model simulations. When simulating trees, we either fix a set of tree model parameters and simulate each tree under the model with the fixed parameters or draw a new set of parameters from prior distributions for each tree. Further we either simulate the process until it reaches a pre-defined number of sampled nodes or until a pre-defined time length (the time of origin) is reached.

For models without ρ-sampling, we fix one of the parameters to its true value in MCMC (as not all parameters can be inferred). In all scenarios, we place a uniform prior on $[0, 1000]$ for the time of origin.

We simulate sequences of 2000 bp under the GTR model with fixed rates and frequencies:

$$(\eta_{AC}, \eta_{AG}, \eta_{AT}, \eta_{CG}, \eta_{CT}, \eta_{GT}) = (0.4, 1.0, 0.1, 0.15, 1.04, 0.15)$$

$$(\pi_A, \pi_C, \pi_G, \pi_T) = (0.25, 0.25, 0.25, 0.25)$$

We use the strict molecular clock model with a fixed substitution rate, μ_s. In the scenarios where not stated explicitly, the substitution rate was fixed to 0.01.

For each estimated parameter, we take the median of its posterior distribution as a point estimate. We calculate the error and relative bias of the median estimate and relative 95% high probability density (HPD) interval width defined below and assess whether the true value is inside the 95% HPD interval.

$$error = \frac{|true\ value - median|}{true\ value},$$

$$relative\ bias = \frac{true\ value - median}{true\ value}, \text{ and}$$

$$relative\ 95\%HPD\ width = \frac{upper - lower}{true\ value},$$

where *upper* and *lower* are the upper and lower bounds of the 95% HPD interval.

Then we summarise the statistics from 100 runs and report the medians of 100 errors, 100 relative biases, and 100 relative 95% HPD widths and the 95% HPD accuracy, which is the number of times when the true value is inside a 95% HPD interval.

Simulation of the sampled ancestor birth-death model

Scenario 1

In scenarios 1.1, 1.2, and 1.4, we simulate under the model without ρ-sampling, i.e., $\rho = 0$. In Scenarios 1.1 and 1.2, parameters are fixed and we stop simulations when a sample of 200 is reached. In Scenario 1.3, we simulate trees on a fixed time interval of t_{or}. We discard trees with too small or too large numbers of sampled nodes. In Scenario 1.4, we draw parameters λ, μ, ψ, and r from uniform prior distributions and simulate trees with 100 sampled nodes. The results of this set of simulations are summarised in Table 2.

Scenario 2

In Scenarios 2.1.1 and 2.1.2, we use the model without ρ-sampling and stop simulations when a sample of 200 is reached. The tree model parameters are fixed. We also analyse the data simulated for Scenario 2.1.1 under the model, where r is fixed to one (Scenario A2.1.1). The results are in Table 3.

In Scenarios 2.2 and 2.3, we use d, ν, and s parameterisation, i.e., we estimate and place priors on parameters

$$d = \lambda - \mu$$
$$\nu = \tfrac{\mu}{\lambda}$$
$$s = \tfrac{\psi}{\mu+\psi}$$

instead of λ, μ and ψ.

In Scenario 2.2, we set $r = 0$ and stop simulations when time $t_{or} = 3.5$ reached. The average number of sampled nodes is 50. We discard trees with less than 5 sampled nodes, and analyse

92 remaining trees. For Scenario B2.2, we remove ψ-sampled sequences and analyse remaining sequences fixing ρ to the truth. The results are in Table 4.

In Scenario 2.3, the tree model parameters are drawn from the prior distributions. The stop simulation condition is when the time of origin reaches 3.0. We discard trees with less than 5 or more than 250 sampled nodes, which constitutes 21% of simulated trees. The average number of sampled nodes in remaining trees is 53. s is fixed in MCMC. The results of Scenarios 2.3 and A2.3 (where r is fixed to one) are shown in Table 5 and Figure 1.

Simulation of the sampled ancestor skyline model

In all three scenarios for simulation of the skyline model, we simulate the process until a sample of 200 is reached. We only simulate trees and do not simulate sequences in these scenarios. In Scenario 1.1, there are two intervals and only sampling rate shifts from zero to non-zero value. In Scenario 1.2, there are two intervals and all parameters except r, which is fixed in the MCMC, shifts at time $t_1 = 5.0$. In Scenario 1.3, we have three intervals with the shift times $t_1 = 3.0$ and $t_2 = 6.0$ and all parameters shift at these times. All elements of vector $\bar{r}$ are fixed in MCMC. In this last scenario, we simulated 50 trees and present the results on 42 successful MCMC runs (other runs did not converge with the chain length of 20M states). The results are in Table 6.

HIV-1 dataset analysis

The accession numbers for some of the taxon names in the tree in Figure 8 in the main text are given in the table.

taxon name	accession number	taxon name	accession number
129717	AY362152.1	103979	AY362145.1
126787	AY362151.1	134795	AY362101.1
102429	AY362149.1	134284	AY362100.1
103176	AY362148.1	101559	AY362057.1
124923	AY362147.1	102536	AY362056.1
117505	AY362146.1	RO1R	AF494119.1

Figures

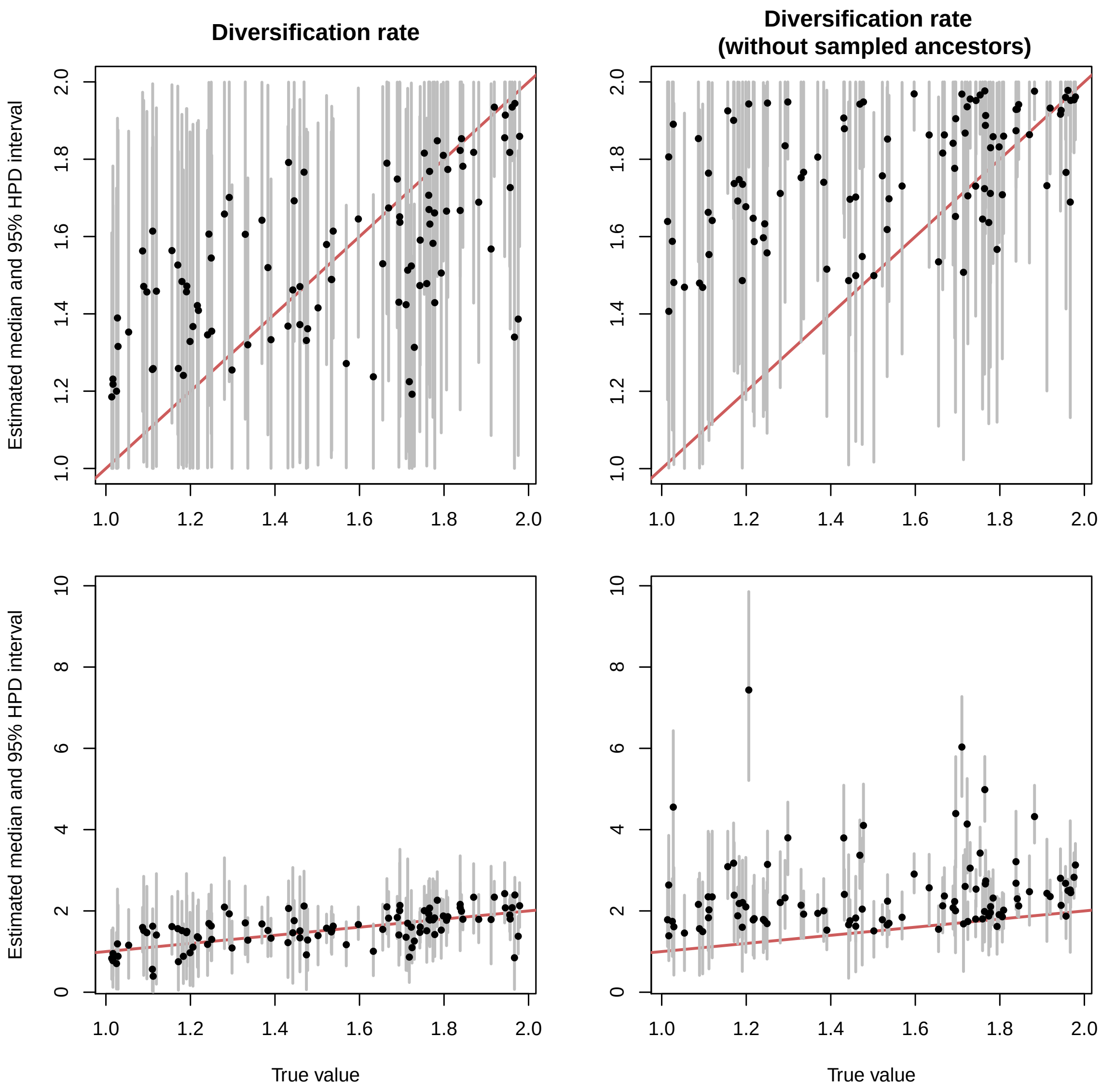

Figure 1: **Diversification rate estimates for the simulated transmission birth-death process.** The graphs in the left column show the estimates from the analyses under the correct model, Scenario 2.3, and in the right column from the analyses under the model without sampled ancestors (misspecified model), Scenario A2.3. The top row corresponds to the analyses where the prior distribution for the diversification rate was Uniform(1,2), which is the same as the distribution from which the rate was drawn for simulations. The bottom row shows the same analyses of the same simulated data but with Uniform(0,1000) prior distribution for the diversification rate. It is well seen on the right graphs that not accounting for sampled ancestors introduces a substantial positive bias.

Large tables

Table 2: **Scenario 1 for the sampled ancestor model.** The table summarises posterior estimates of parameters and accuracy obtained from analyses of simulated trees.

	true value	prior	median	error	relative bias	relative 95% HPD width	95% HPD accuracy in %
Scenario 1.1: ψ fixed in MCMC.							
λ	0.9	U(0,100)	0.9222	0.0735	0.0247	0.4056	96
μ	0.2	U(0,100)	0.2219	0.3885	0.1096	2.2460	94
ψ (fixed)	0.3	-	-	-	-	-	-
r	0.6	U(0,1)	0.5823	0.0880	-0.0295	0.4087	93
Scenario 1.2: r fixed in MCMC.							
λ	1.0	U(0,100)	1.0861	0.0921	0.0861	0.5020	96
μ	0.1	U(0,100)	0.1871	0.8705	0.8705	4.8774	98
ψ	0.4	U(0,100)	0.3841	0.0888	-0.0399	0.4821	93
r (fixed)	0.5	-	-	-	-	-	-
Scenario 1.3: $r = 0$ and simulations stop at t_{or}.							
t_{or}	5.0	U(0,1000)	4.8545	0.0815	-0.0291	0.4509	97
λ	1.5	U(0,100)	1.6077	0.1112	0.0718	0.7094	93
μ	0.5	U(0,100)	0.6494	0.4088	0.2988	2.1971	94
ψ	0.2	U(0,100)	0.1884	0.1840	-0.0579	0.8871	90
ρ	0.8	U(0,1)	0.7756	0.0916	-0.0305	0.5446	97
Scenario 1.4: parameters drawn from priors.							
λ	-	U(1,1.5)	1.2899	0.0640	-0.0106	0.3324	95
μ	-	U(0.5, 1)	0.6727	0.1546	0.0321	0.6520	92
ψ (fixed)	-	U(4,5)	-	-	-	-	-
r	-	U(0,1)	0.0499	0.4630	-0.0853	2.0262	92

Table 3: **Scenario 2 for the sampled ancestor model.** The table summarises posterior estimates of parameters and tree properties and accuracy obtained from analyses of simulated sequence data. Tree model parameters were fixed for simulation.

	true value	prior	median	error	relative bias	relative 95% HPD width	95% HPD accuracy in %
Scenario 2.1.1: $\mu_s = 0.02$.							
t_{or}	-	U(0,1000)	-	0.0347	0.0066	0.2398	95
t_{root}	-	SABD[2]	-	0.0065	-1e05	0.0404	97
# SA	-	SABD[2]	-	0.0714[3]	0.0000[3]	0.3043[3]	97
λ	1.0	U(0,100)	1.0486	0.0825	0.0486	0.4231	93
μ	0.2	U(0, 100)	0.2456	0.3436	0.2279	2.5473	95
ψ (fixed)	0.4	-	-	-	-	-	-
r	0.7	U(0,1)	0.6753	0.057	-0.0353	0.3301	93
Scenario A2.1.1: the same as 2.1.1 but r is fixed to one.							
t_{or}	-	U(0,1000)	-	0.0332	0.0042	0.2191	94
t_{root}	-	SABD[2]	-	0.0072	0.0029	0.0413	96
λ	1.0	U(0,100)	1.2063	0.2063	0.2063	0.4854	55
μ	0.2	U(0, 100)	0.2996	0.5350	0.4982	2.9346	91
ψ (fixed)	0.4	-	-	-	-	-	-
r (fixed)	0.7	1.0	-	-	-	-	-
Scenario 2.1.2: $\mu_s = 0.0002$.							
t_{or}	-	U(0,1000)	-	0.0504	0.0009	0.2856	94
t_{root}	-	SABD[2]	-	0.0221	-0.0067	0.1423	96
# SA	-	SABD[2]	-	0.3784[3]	0.2660[3]	1.9307[3]	98
λ	1.0	U(0,100)	1.1206	0.1343	0.1206	0.7940	95
μ	0.2	U(0,100)	0.3397	0.6984	0.6984	4.0758	95
ψ (fixed)	0.4	-	-	-	-	-	-
r	0.7	U(0,1)	0.5726	0.1915	-0.1820	1.0405	93

[2]SABD stands for sampled ancestor birth-death model.

[3]To calculate errors, relative biases and relative HPD widths for #SA we increased true value, median estimate and lower and upper HPD estimates by one because the relative statistics are not defined if a true value is equal to zero.

Table 4: **Scenario 2 for the fossilised birth-death model.** The table summarises posterior estimates of parameter and tree properties and accuracy obtained from analyses of simulated sequence data. Tree model parameters were fixed for simulation.

	true value	prior[2]	median	error	relative bias	relative 95% HPD width	95% HPD accuracy in %
Scenario 2.2: $r = 0$ and simulations stop at t_{or}.							
t_{or}	3.5	U(0,1000)	3.5776	0.0857	0.0222	0.5816	96
t_{root}	-	SABD[2]	-	0.0170	0.0000	0.1480	95
# SA	-	SABD[2]	-	0.0241[3]	0.0000[3]	0.1905[3]	99
μ_s	0.01	ln$\mathcal{N}$(-4.6, 1.25)[4]	0.0099	0.0342	-0.0076	0.2304	95
d	1.0	U(0,1000)	1.0266	0.1872	0.0266	1.0317	95
ν	0.3333	U(0,1)	0.3343	0.2236	0.0029	1.7816	100
s	0.4444	U(0,1)	0.4343	0.1844	-0.0229	1.2984	98
ρ	0.7	U(0,1)	0.6854	0.1116	-0.0209	0.8005	95
Scenario B2.2: the same as 2.2 but without ψ-sampled sequences and with ρ fixed.							
t_{or}	3.5	U(0,1000)	3.4743	0.1442	-0.0073	0.7990	93
t_{root}	-	SABD[2]	-	0.0891	-0.0255	0.5169	95
# SA	-	SABD[2]	-	0.1111	0.0000	0.6364	99
μ_s	0.01	ln$\mathcal{N}$(-4.6, 1.25)[4]	0.0099	0.0342	-0.0076	0.2304	95
d	1.0	U(0,1000)	0.9532	0.2403	-0.0468	1.2044	98
ν	0.3333	U(0,1)	0.3361	0.2064	0.0084	2.0467	100
s	0.4444	U(0,1)	0.4564	0.1557	0.0269	1.6076	100
ρ (fixed)	0.7	-	-	-	-	-	-

[4]ln$\mathcal{N}(\alpha, \beta)$ is a Log-normal distribution with mean α and standard deviation β in the log-transformed space.

Table 5: **Scenario 2 for the transmission birth-death model.** The table summarises posterior estimates of parameters and tree properties and accuracy obtained from analyses of simulated sequence data. Tree model parameters were drawn from the prior distributions.

	true value	prior[2]	median	error	relative bias	relative 95% HPD width	95% HPD accuracy in %
Scenario 2.3: $\rho = 0$, parameters drawn from priors and simulations stop at t_{or}.							
t_{or}	3.0	U(0,1000)	3.0084	0.05096	0.0157	0.4301	98
t_{root}	-	SABD[2]	-	0.0168	-1e-07	0.1322	94
# SA	-	SABD[2]	-	0.0493[3]	0.0000[3]	0.3636[3]	98
μ_s	0.01	ln$\mathcal{N}$(-4.6, 1.25)[4]	0.0100	0.0550	0.0023	0.2515	93
d	-	U(1,2)	1.5217	0.1053	0.0023	0.5239	93
ν	-	U(0,1)	0.1956	0.2084	0.0037	0.8309	95
r	-	U(0,1)	0.3146	0.2814	-0.0065	1.3304	92
s (fixed)	-	U(0.5, 1)	-	-	-	-	
Scenario A2.3: the same as 2.3 but r is fixed to one.							
t_{or}	3.0	U(0,1000)	3.0518	0.0566	0.0263	0.4040	98
t_{root}	-	SABD[2]	-	0.0218	0.0113	0.1383	82
μ_s	0.01	ln$\mathcal{N}$(-4.6, 1.25)[4]	0.0094	0.0667	-0.0556	0.2533	69
d	-	U(1,2)	1.7662	0.1250	0.1224	0.3937	69
ν	-	U(0,1)	0.1875	0.2197	-0.0287	0.7362	85
r (fixed)	U(0,1)	1.0	-	-	-	-	-
s (fixed)	-	U(0.5, 1)	-	-	-	-	

Table 6: **Scenario 1 for the sampled ancestor skyline model.** The table summarises posterior parameter estimates and accuracy obtained from analyses of simulated trees.

	true value	prior	median	error	relative bias	relative 95% HPD width	95% HPD accuracy in %
Scenario 1.1: two intervals and only ψ shifts from zero to non-zero value.							
λ	0.8	U(0,100)	0.8107	0.0861	0.0134	0.4577	92
μ	0.4	U(0,100)	0.4199	0.2070	0.0499	1.1330	94
ψ (fixed)	0.2	-	-	-	-	-	-
r	0.8	Uniform(0,1)	0.7874	0.0394	-0.0158	0.2516	97
Scenario 1.2: two intervals and all parameters except r shift.							
λ_1	1.0	U(0, 100)	1.1869	0.2072	0.1869	1.1345	100
λ_2	0.8	U(0, 100)	0.8442	0.065	0.0552	0.6402	100
μ_1	0.2	U(0, 100)	0.3660	0.8298	0.8298	6.0261	100
μ_2	0.2	U(0, 100)	0.2640	0.4035	0.3200	3.1496	100
ψ_1	0.4	U(0, 100)	0.3452	0.2056	-0.1371	0.9341	94
ψ_2	0.5	U(0, 100)	0.4847	0.0915	-0.0305	0.5592	96
r (fixed)	0.7	-	-	-	-	-	-
Scenario 1.3: three intervals, all parameters shift and vector $\bar{r}$ fixed.							
λ_1	1.5	U(0, 100)	1.6988	0.2385	0.1325	1.2336	95
λ_2	1.2	U(0, 100)	1.3945	0.2014	0.1621	0.8813	95
λ_3	0.5	U(0, 100)	0.5480	0.1568	0.0960	1.2625	100
μ_1	0.5	U(0, 100)	0.7108	0.5132	0.4216	3.5732	100
μ_2	0.6	U(0, 100)	0.8086	0.4067	0.3477	1.9862	90
μ_3	0.2	U(0, 100)	0.2594	0.4856	0.2968	3.2805	100
ψ_1	0.4	U(0, 100)	0.4057	0.2359	0.0141	1.2262	90
ψ_2	0.5	U(0, 100)	0.4497	0.1706	-0.1006	0.6650	90
ψ_3	0.1	U(0, 100)	0.0967	0.1933	-0.0327	1.0046	98
r_1 (fixed)	0.1	-	-	-	-	-	-
r_2 (fixed)	0.5	-	-	-	-	-	-
r_3 (fixed)	0.9	-	-	-	-	-	-
